# Supplementary material for: Multifaceted and Intricate Oncogenic Mechanisms of NDRG1 in Head and Neck Cancer Depend on Its C-Terminal 3R-Motif
Source: Cells. 2022 May 7;11(9):1581. doi: 10.3390/cells11091581 (PMC9104279; doi:10.3390/cells11091581)
Supplement: Supplementary file 1 [file cells-11-01581-s001.zip › cells-1683959-supplementary.pdf]

Supplementary Material:

Multifaceted and intricate oncogenic mechanisms of NDRG1 in head and neck cancer depend on its C-terminal 3R-motif

Guo-Rung You, Joseph T. Chang, Hsiao-Fan Li, Ann-Joy Cheng

Figure S1

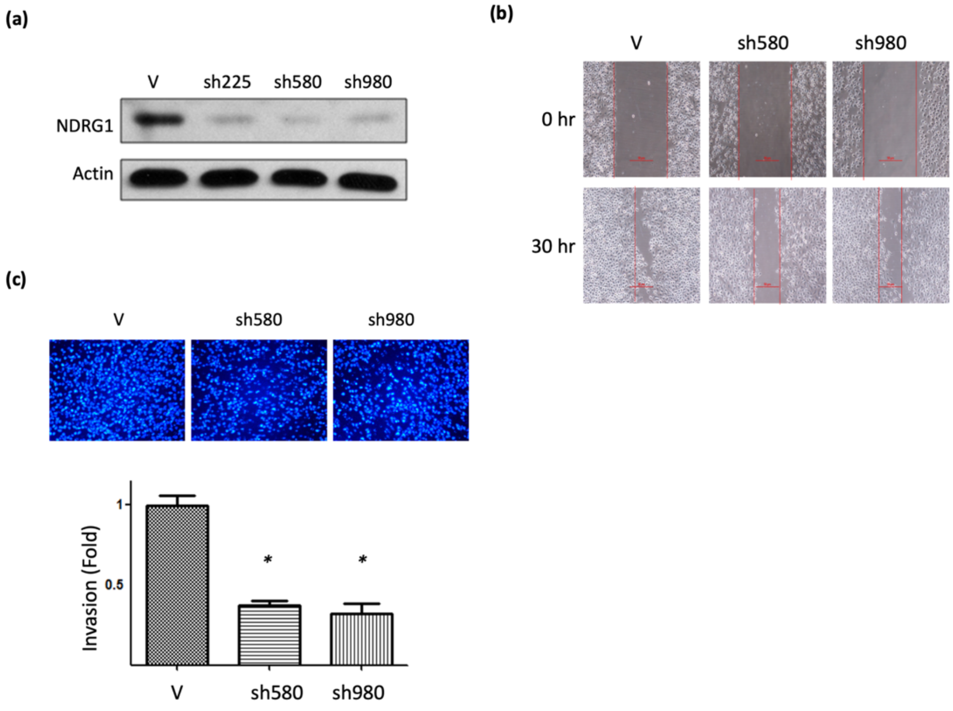

Figure S1. Silencing NDRG1 inhibited cell migration and invasion in OECM1 cells. (**a**) Knockdown efficiency of NDRG1-shRNA in OECM1 cells. V: pGSH1-GFP, sh225: pGSH1-sh225, pGSH1-sh580, pGSH1-sh980 (Specific NDRG1-shRNA oligonucleotides were be cloned into pGSH1-GFP vector. (pGSH1-GFP-sh225: 5'-gau ccg agu uug aug ucc agg agc gaagcuug gcu ccu gga cau caa acu cgg auc uu-3'; pGSH1-GFP-sh580: 5'-gau ccc tcg auu ugc ucu aaa caa gaagcuug uug uuu aga gca aau cga agg gau cuu-3', pGSH1-GFP-sh980: 5'-gau ccg aga cca cuc ucc uca aga gaagcuug ucu uga gga gag ugg ucu cgg auc uu-3'). (**b**) Silencing NDRG1 in OECM1 cells decreased the cell motility, as determined by the wound-healing migration assay. (**c**) Silencing NDRG1 in OECM1 cells inhibited cell invasion behavior, as determined by Matrigel

invasion assay. The error bars shown in the relevant figures indicated the standard deviation of the quantification results in all experiments. (* $p < 0.05$; *t*-test).

Table S1. List of primers used for RT-qPCR analysis.

Gene	Primer Sequences (5’ -> 3’)
Twist1	F: GAGTCCGCAGTCTTACGAGG
	R: CTGCCCGTCTGGGAATCACT
BMI1	F: GCTGCCAATGGCTCTAATGAA
	R: TGCTGGGCATCGTAAGTATCTT
NES	F: CTGCTACCCTTGAGACACCTG
	R: GGGCTCTGATCTCTGCATCTAC
c-Met	F: AGCAATGGGGAGTGTAAAGAGG
	R: CCCAGTCTTGTACTCAGCAAC
ABCG2	F: CAGGTGGAGGCAAATCTTCGT
	R: ACCCTGTTAATCCGTTCGTTTT
Fibronectin	F: GGTGACACTTATGAGCGTCCTAAA
	R: TACAATCTACCATCATCCAGCCT
Vimentin	F: TGGCACGTCTTGACCTTGAA
	R: GGTCATCGTGATGCTGAGAA
N-cadherin	F: GACAATGCCCCTCAAGTGTT
	R: CCATTAAGCCGAGTGATGGT

Table S2. List of antibodies used for western blot studies.

Primary antibody	Provider	Species	Reference
NDRG1	abcam	Mouse	ab56697
GAPDH	EMD Millipore	Mouse	MAB374
CD44	Agent	Mouse	AM1901b
Twist1	Santa Cruz	Rabbit	SC-15393
Fibronectin	Santa Cruz	Mouse	SC-18825
Vimentin	abcam	Rabbit	ab8978
Phosphoserine	abcam	Mouse	ab6639
GFP	abcam	Mouse	ab290
HDAC1	Upstate	Mouse	05-614
**Secondary antibody**	**Provider**	**Species**	**Reference**
Goat anti-mouse IgG-HRP	Santa Cruz	Goat	SC-2005
Anti-rabbit IgG HRP-linked	Cell signaling	Goat	7074

Table S3. Top 50 up-regulated and top 50 down-regulated genes in NDRG1 over-expressing OECM1 cells.

Type	Gene Symbol	Description	Fold Change
Up	C9orf64	chromosome 9 open reading frame 64	28.078
Up	EIF1AY	eukaryotic translation initiation factor 1A, Y-linked	19.534
Up	FLRT3	fibronectin leucine rich transmembrane protein 3	16.941
Up	CALB1	calbindin 1	15.960
Up	CYorf15B	chromosome Y open reading frame 15B	14.659
Up	CASP14	caspase 14	11.447
Up	DAPK1	death-associated protein kinase 1	10.876
Up	SLC47A1	solute carrier family 47, member 1	7.007
Up	KCNMA1	potassium large conductance calcium-activated channel	6.965
Up	CTSH	cathepsin H	6.850
Up	LOC440173	hypothetical LOC440173	6.751
Up	TNC	tenascin C	6.707
Up	MCART1	mitochondrial carrier triple repeat 1	6.203
Up	HCLS1	hematopoietic cell-specific Lyn substrate 1	5.751
Up	NLGN4Y	neuroligin 4, Y-linked	5.492
Up	ADRBK2	adrenergic, beta, receptor kinase 2	5.476
Up	TRIB2	tribbles homolog 2	5.353
Up	CIT	citron	5.333
Up	TIMP3	TIMP metallopeptidase inhibitor 3	4.930
Up	RRM2	ribonucleotide reductase M2	4.867
Up	MLF1	myeloid leukemia factor 1	4.846
Up	KNTC1	kinetochore associated 1	4.758
Up	CLSPN	claspin homolog	4.740
Up	MCM10	minichromosome maintenance complex component 10	4.622
Up	FAM58A	family with sequence similarity 58	4.605
Up	MCM8	minichromosome maintenance complex component 8	4.507
Up	ETV5	ets variant 5	4.397
Up	CKS2	CDC28 protein kinase regulatory subunit 2	4.393
Up	NAT5	N-acetyltransferase 5	4.354
Up	CDC6	cell division cycle 6	4.334
Up	DPYSL3	dihydropyrimidinase-like 3	4.251
Up	FANCA	Fanconi anemia	4.241
Up	SHCBP1	SHC SH2-domain binding protein 1	4.152
Up	SULF2	sulfatase 2	4.085
Up	FAM111B	family with sequence similarity 111, member B	4.077

Up	SLC23A2	solute carrier family 23	4.076
Up	RPS27L	ribosomal protein S27-like	4.060
Up	PBK	PDZ binding kinase	3.984
Up	C11orf82	chromosome 11 open reading frame 82	3.983
Up	CHST10	carbohydrate sulfotransferase 10	3.973
Up	CHEK1	CHK1 checkpoint homolog	3.925
Up	KCNJ2	potassium inwardly-rectifying channel	3.881
Up	PCNA	proliferating cell nuclear antigen	3.792
Up	MKKS	McKusick-Kaufman syndrome	3.761
Up	RNASEH2A	ribonuclease H2, subunit A	3.705
Up	WDHD1	WD repeat and HMG-box DNA binding protein 1	3.691
Up	PDZK1IP1	PDZK1 interacting protein 1	3.678
Up	TSHZ2	teashirt zinc finger homeobox 2	3.674
Up	UNQ5815	UNQ5815	3.672
Up	C20orf72	chromosome 20 open reading frame 72	3.623
Down	OAS2	2'-5'-oligoadenylate synthetase 2	-39.198
Down	STARD4	StAR-related lipid transfer domain containing 4	-22.281
Down	MPPED2	metallophosphoesterase domain containing 2	-20.748
Down	GBP4	guanylate binding protein 4	-15.588
Down	IL7R	interleukin 7 receptor	-14.839
Down	TRIM31	tripartite motif-containing 31	-13.282
Down	LIPH	lipase, member H	-12.233
Down	MX2	myxovirus resistance 2	-12.106
Down	GBP1	guanylate binding protein 1	-11.326
Down	STRA6	stimulated by retinoic acid gene 6	-10.255
Down	GCNT4	glucosaminyl (N-acetyl) transferase 4	-9.631
Down	TRIM22	tripartite motif-containing 22	-9.317
Down	SLC16A14	solute carrier family 16	-9.164
Down	BMP5	bone morphogenetic protein 5	-9.061
Down	PSG9	pregnancy specific beta-1-glycoprotein 9	-8.860
Down	AIM2	absent in melanoma 2	-8.859
Down	ZNF91	zinc finger protein 91	-8.087
Down	UBA7	ubiquitin-like modifier activating enzyme 7	-8.069
Down	HERC5	hect domain and RLD 5	-8.024
Down	SAMD9L	sterile alpha motif domain containing 9-like	-7.845
Down	KRT13	keratin 13	-7.813
Down	MDK	midkine	-7.708
Down	LOXL4	lysyl oxidase-like 4	-7.252

Down	TMEM156	transmembrane protein 156	-6.960
Down	CDH10	cadherin 10, type 2	-6.906
Down	SEMA3E	sema domain	-6.647
Down	TET1	tet oncogene 1	-6.521
Down	ZNF675	zinc finger protein 675	-6.428
Down	SCNN1A	sodium channel, nonvoltage-gated 1 alpha	-6.371
Down	CD24	CD24 molecule	-6.248
Down	PSMB9	proteasome subunit, beta type, 9	-6.247
Down	IRF1	interferon regulatory factor 1	-6.179
Down	FLJ13744	hypothetical FLJ13744	-5.986
Down	SYT14	synaptotagmin XIV	-5.885
Down	PARP14	poly (ADP-ribose) polymerase family, member 14	-5.865
Down	PLAU	plasminogen activator, urokinase	-5.863
Down	IFI44	interferon-induced protein 44	-5.667
Down	CPN1	carboxypeptidase N, polypeptide 1	-5.599
Down	GRAMD1C	carboxypeptidase N, polypeptide 1	-5.562
Down	SLC2A12	solute carrier family 2	-5.507
Down	SERPINB1	serpin peptidase inhibitor,clade B	-5.452
Down	PLAC8	placenta-specific 8	-5.426
Down	IFIH1	interferon induced with helicase C domain 1	-5.417
Down	SLC6A9	solute carrier family 6	-5.414
Down	PPP1R12B	protein phosphatase 1, regulatory subunit 1	-5.414
Down	ANKRD36B	ankyrin repeat domain 36B	-5.361
Down	TRIML2	tripartite motif family-like 2	-5.228
Down	LPHN2	latrophilin 2	-5.209
Down	C4orf33	chromosome 4 open reading frame 33	-5.189
Down	CLEC7A	C-type lectin domain family 7	-5.170
